# Combining Image Restoration and Traction Force Microscopy to Study Extracellular Matrix-Dependent Keratin Filament Network Plasticity

**DOI:** 10.3389/fcell.2022.901038

**Published:** 2022-05-11

**Authors:** Sungjun Yoon, Reinhard Windoffer, Aleksandra N. Kozyrina, Teodora Piskova, Jacopo Di Russo, Rudolf E. Leube

**Affiliations:** ^1^ Institute of Molecular and Cellular Anatomy, RWTH Aachen University, Aachen, Germany; ^2^ Interdisciplinary Centre for Clinical Research, RWTH Aachen University, Aachen, Germany; ^3^ DWI-Leibniz-Institute for Interactive Materials Forckenbeckstr, Aachen, Germany

**Keywords:** cytoskeleton, intermediate filaments, keratin, image restoration, traction force microscopy, extracellular matrix

## Abstract

Keratin intermediate filaments are dynamic cytoskeletal components that are responsible for tuning the mechanical properties of epithelial tissues. Although it is known that keratin filaments (KFs) are able to sense and respond to changes in the physicochemical properties of the local niche, a direct correlation of the dynamic three-dimensional network structure at the single filament level with the microenvironment has not been possible. Using conventional approaches, we find that keratin flow rates are dependent on extracellular matrix (ECM) composition but are unable to resolve KF network organization at the single filament level in relation to force patterns. We therefore developed a novel method that combines a machine learning-based image restoration technique and traction force microscopy to decipher the fine details of KF network properties in living cells grown on defined ECM patterns. Our approach utilizes Content-Aware Image Restoration (CARE) to enhance the temporal resolution of confocal fluorescence microscopy by at least five fold while preserving the spatial resolution required for accurate extraction of KF network structure at the single KF/KF bundle level. The restored images are used to segment the KF network, allowing numerical analyses of its local properties. We show that these tools can be used to study the impact of ECM composition and local mechanical perturbations on KF network properties and corresponding traction force patterns in size-controlled keratinocyte assemblies. We were thus able to detect increased curvature but not length of KFs on laminin-322 versus fibronectin. Photoablation of single cells in microprinted circular quadruplets revealed surprisingly little but still significant changes in KF segment length and curvature that were paralleled by an overall reduction in traction forces without affecting global network orientation in the modified cell groups irrespective of the ECM coating. Single cell analyses furthermore revealed differential responses to the photoablation that were less pronounced on laminin-332 than on fibronectin. The obtained results illustrate the feasibility of combining multiple techniques for multimodal monitoring and thereby provide, for the first time, a direct comparison between the changes in KF network organization at the single filament level and local force distribution in defined paradigms.

## Introduction

Cytoplasmic intermediate filaments (IFs) are prominent components of the cytoskeleton forming complex 3D networks, which interlink cellular organelles, adjacent cells and the extracellular matrix [ECM; (Schwarz and Leube, 2016; Broussard et al., 2020; Redmond and Coulombe, 2021)]. The term “intermediate” was coined because of their diameter (∼10 nm), which is in between that of the other major cytoskeletal filament systems, i.e. the actin-based microfilaments (∼7 nm) and tubulin-based microtubules (∼25 nm). IFs are considered to be highly stable with limited motility and high resistance against physical and chemical stresses (Pekny and Lane, 2007; van Bodegraven and Etienne-Manneville, 2021). They are compositionally diverse participating in most cellular processes and performing important mechanical functions (Block et al., 2015; Hatzfeld et al., 2017; Etienne-Manneville, 2018).

The epithelial keratin intermediate filaments (KFs) are by far the most complex IF type. They are encoded by two multigene families, which encompass 28 type I and 26 type II keratin polypeptides in human (Loschke et al., 2015; Jacob et al., 2018). Recent studies have emphasized that KFs are highly dynamic structures with varying turnover and cell type-specific organization (Iwatsuki and Suda, 2010; Windoffer et al., 2011; Quinlan et al., 2017; Tateishi et al., 2017; Pora et al., 2020). The highly intricate subcellular 3D network arrangement poses additional, difficult-to-address challenges, which necessitate high-resolution network analyses (Nahidiazar et al., 2015; Quinlan et al., 2017; Moch et al., 2020; Moch and Leube, 2021; Prechova et al., 2022; Windoffer et al., 2022). Correlating the subcellular organization with specific functions and changes of the microenvironment has been difficult to accomplish. But such multimodal information is needed to elucidate mechanisms of IF-determined cell behavior in different paradigms.

The aforementioned technical difficulties may—at least in part—be the reason why contradicting results have been reported in the past, notably in cell migration (Yoon and Leube, 2019). For example, some studies have reported that reduced keratin expression leads to decreased cell migration (Bordeleau et al., 2012; Khapare et al., 2012; Iyer et al., 2013; Zhang et al., 2016; Fang et al., 2017; Seltmann et al., 2013; Seltmann et al., 2015), whereas others reported that it leads to increased cell migration (Busch et al., 2012; Fortier et al., 2013; Seltmann et al., 2013; Seltmann et al., 2015). Interestingly, the motility (i.e., flow rate) of keratins was shown to correlate with the speed of migration and the density and elasticity of the ECM (Pora et al., 2020). Further studies revealed the importance of laininin-332-dependent hemidesmosomal β4 integrin clustering for cell spreading and migration (Pora et al., 2019). Details of ECM-dependent KF network organization, however, were difficult to resolve. To study the cross-talk between KFs and the ECM it is necessary to monitor directly *in vivo* how subcellular network dynamics and KF morphology are determined by ECM composition. It is furthermore crucial to link these properties to functional readouts such as local force distribution. To date, studies that relate KF organization with mechanical force depict only the changes in static conditions (Latorre et al., 2018; Fujiwara et al., 2020; Karsch et al., 2020; Prechova et al., 2022) since methods for quantitative analyses of KF dynamics in relation to cell mechanics have not been available.

Here, we describe methods which utilize machine learning-based image restoration for enhanced spatial resolution. They are used to quantitatively analyze the structural properties of KFs enabling not only the correlation with ECM composition but also enabling assessment of local force distribution by traction force measurements under standardized growth conditions. This high resolution multimodal approach was exemplarily applied to examine the effect of photoablating single cells in small motile cell groups. We show that subcellular resolution can be obtained with the combination of the different techniques to elucidate structure-function relationships of the KF cytoskeleton in defined microenvironments.

## Materials and Methods

### Cell Culture

Immortalized human HaCaT B10 keratinocytes expressing EYFP-tagged human keratin 5 (HK5-EYFP) were recently described (Moch et al., 2013). The cells were grown at 37°C in a 5% CO_2_ humidified incubator. They were maintained in Dulbeccos’s Modified Eagle’s Medium (DMEM) containing l-alanyl-glutamine (Sigma-Aldrich) and 10% (v/v) fetal bovine serum (SeraPlus; PAN Biotech). For passaging, cells were first washed and incubated for 15 min with PBS without Ca^++^/Mg^++^ (Sigma-Aldrich) to destabilize desmosomes and were thereafter trypsinized for 3 min in PBS without Ca^++^/Mg^++^ containing 0.25% (w/v) trypsin (Biochrom) supplemented with 0.02% (w/v) EDTA (Sigma-Aldrich). The detached cells were then centrifuged (1,000 rpm for 3 min) and resuspended in fresh medium. Cells were passaged once every 7–9 days, i.e. 1-2 days after reaching confluence.

### Micropatterning of Glass Coverslips

The entire process of micropatterning glass coverslips is summarized in Supplementary Figure S1. It consisted of multiple steps:

#### Poly(L-Lysine)-Graft-Poly(Ethylene Glycol) (pLL-g-PEG) Glass Coating

Eighteen millimeter glass coverslips were washed with isopropanol and sonicated for 5 min to completely remove dust particles. The coverslips were then placed in a plasma cleaner horizontally with the ECM coating side facing upwards. The plasma was run for 15 s at 30 W with a gas flow rate of 5 ml/min (sscm). A drop of poly(L-lysine)-graft-poly(ethylene glycol) solution [pLL-g-PEG (SuSoS; 0.1 mg/ml in 10 mM 4-(2-hydroxyethyl)-1-piperazineethanesulfonic acid (HEPES)] was placed on parafilm, and the coverslip was placed on top of the solution with the plasma-treated side towards the solution. After incubating for 30 min at room temperature, coverslips were gently lifted off and placed on holders vertically to let the remaining solution run off by gravity. The treated coverslips were either used immediately afterwards or stored at 4°C.

#### Deep UV Insolation

Photomasks (custom designed by Compugraphics) were first cleaned with 1 ml of isopropanol and exposed to ultraviolet light (UV) using the UVO-Cleaner from Jelight Company for 5 min without the pLL-g-PEG coated coverslip. After UV exposure, 5 μl of milliQ water was placed between the photomask and the coated coverslip to act as a temporary adhesive. With the coverslip side facing down, the photomask was then exposed to UV for additional 8 min to detach the PEG chain and allow protein binding only on the UV-exposed surface. After removing the photomask from the UVO-Cleaner, 1 ml of milliQ water was placed around the coverslip to allow detachment. The detached coverslip was then briefly washed in PBS and was either used immediately afterwards or stored at 4°C.

#### ECM Coating

ECM proteins of interest were coated on the UV-activated sites of the coverslips in this step. A drop of either human fibronectin solution (VWR; 20 μg/ml in 100 mM NaHCO_3_) or laminin-332 (Sigma-Aldrich; 10 μg/ml in 100 mM NaHCO_3_) was placed on parafilm. Coverslips were then placed on top of the protein solution droplets with the coated side facing the solution and incubated for 1 h at room temperature. The resulting micropatterned ECM-coated coverslips were briefly washed in PBS and used immediately afterwards for either direct cell seeding or transferred onto the polyacrylamide gel.

### Preparation of Micro-patterned ECM Substrates on Polyacrylamide Hydrogels

The objective of this step was to prepare an elastic hydrogel substrate with micropatterned ECM on a glass-bottom culture dish.

To this end, the glass of 35 mm petri dishes with 20 mm glass-bottoms (Cell E&G) were first silanized to ensure stable attachment to the polyacrylamide gel. Silanization was carried out by incubating the glass for 5 min in 0.1 M NaOH, washing it with ddH_2_O, incubating it for 5 min in 4% (v/v) 3-aminopropyltriethoxysilane (APTS; Sigma-Aldrich) in isopropanol, washing with ddH_2_O, incubating for 30 min in 1% (v/v) glutaraldehyde (Bio-Rad) in ddH_2_O, washing with ddH_2_O and drying. Both APTS and glutaraldehyde solutions were freshly prepared before silanization.

The polyacrylamide hydrogel was prepared next. For all experiments, 11 kPa stiffness polyacrylamide hydrogels were used. Hydrogels were formed by preparing a mixture of 10% acrylamide (Bio-Rad), 0.07% bis-acrylamide (Bio-Rad), 0.003% tetramethylethylenediamine (Bio-Rad), 0.03% ammonium persulfate (Sigma) and 0.01% carboxy-modified polystyrene beads (Invitrogen F8800) diluted in PBS (Fisher). Immediately after mixing the components, 57 μl of the acrylamide solution was placed on top of the ECM-coated coverslips, which were then covered with silanized glass-bottom dishes, sandwiching the solution between the glasses. The polyacrylamide solution was allowed to polymerize for 30 min at room temperature. After polymerization, coverslips were gently lifted off and cells were seeded on the hydrogel immediately afterwards.

### Immunofluorescence

HaCaT B10 cells were seeded on 18 mm diameter, high-precision glass cover slips with 170 μm thickness (Paul Marienfeld) that were coated with ECM proteins at a density of 10,000 cells/cm^2^ in six-well dishes (CytoOne). After 24 h incubation, cells were fixed with 4% (w/v) paraformaldehyde (PFA) in PBS for 10 min at room temperature and subsequently permeabilized in 0.2% (v/v) Triton-X-100 (Sigma-Aldrich) in PBS for additional 10 min. Prior to fixation, the PFA solution was warmed to 37°C. For the staining procedure, cells were first incubated with 5% (w/v) bovine serum albumin (BSA, SERVA Electrophoresis) in PBS for 30 min at room temperature. Cells were then incubated with the primary antibodies suspended in 1% BSA/PBS solution for 90 min at room temperature, followed by three washing steps of 5 min each in PBS. After washing, cells were incubated with the secondary antibodies suspended in 1% BSA/PBS solution for 30 min at room temperature, followed by three 5 min washing steps in PBS and one 5 min washing step in ddH_2_O. When Alexa-555 phalloidin (Invitrogen) was used, it was mixed with the primary antibodies. Cells were then mounted with Mowiol (Carl Roth) on glass slides. The prepared samples were dried overnight at 4°C before imaging.

The following primary antibodies were used: anti-paxillin (clone 349, mouse; BD Biosciences 610051) and anti-integrin β4 (CD104 clone 439-9B, rat; BD Pharmingen 55719). The following secondary antibodies were used: Alexa-633 goat anti-mouse (Invitrogen A-21053) and DyLight TM 405 donkey anti-rat (Jackson-Dianova 712-476-153).

### Imaging Conditions

Structured illumination fluorescence microscopy was performed with an ApoTome.2 microscope (Zeiss) equipped with an oil immersion objective 63× (N.A. 1.4, DIC, Plan apochromat).

Live-cell imaging and database construction for image enhancement were performed with a laser scanning confocal microscope (LSM 710; Carl Zeiss) using Zen software (black edition 2.1 SP3; Carl Zeiss). The microscope is equipped with an Airyscan detector, a water immersion objective (40×/1.40-N.A. DIC M27) and a focus-shift correction system (DefiniteFocus; all from Carl Zeiss). For live-cell imaging, the microscope was pre-warmed to 37°C and a 5% CO_2_ humidified atmosphere was used. Prior to live cell imaging, the cell culture medium was replaced by 25 mM HEPES-buffered DMEM without phenol red (Life Technologies) supplemented with 2% fetal calf serum.

For photo-oxidation-induced cell lysis, cells were incubated with 2 μg/ml Hoechst 33342 for 30 min prior to imaging. To induce photo-oxidation, cell nuclei of interest were then bleached using the 405 nm diode laser at maximum power intensity and the cells were incubated for additional 20 min before subsequent image acquisition.

### Image Analysis

The following procedures were performed to enable automated image analysis:

#### Keratin Flow Analysis

To analyze keratin dynamics, HaCaT B10 cells were grown on ECM-coated D-shaped micropatterns to mimic polarized migratory behavior in a standardized format. Time-lapse fluorescence recordings of 10 focal planes were acquired every min for 30 min. The resulting image series were analyzed using the CMove program (Pora et al., 2020).

#### Measurement of Rotational Migration

To analyze the rotational movement of keratinocytes, HaCaT B10 cells were grown on circular micropatterns with 47 μm diameter. 24 h after seeding, time-lapse fluorescence recordings were carried out (every 3 min for 1 h). Thirty eight image series were analyzed for each experimental condition using the particle image velocimetry (PIV)-based Fiji angle tool to measure the degree of rotation within the imaging time frame.

### Enhancement of 3D Keratin Filament Network Recordings

To improve the quality of 3D keratin network recordings and to compensate for the limited acquisition time, Content-Aware Image Restoration (CARE) technique was used as described (Weigert et al., 2018). Briefly, low quality images were acquired using fast acquisition settings and compared to matching long acquisition settings as “ground truth” to train a restoration model. After validating the reliability of this model, it was used to denoise experimental live-cell images, which were acquired with reduced laser power and exposure time. For the purpose of this research, the CARE model was used for 3D denoising of the keratin network. The trained restoration model is provided in Supplementary File S1.

### Traction Force Microscopy

Traction force microscopy was performed as described (Aratyn-Schaus et al., 2010) using the above described hydrogel substrate. After each imaging experiment, cells were trypsinized in the incubation chamber for 15 min and the resulting bead positions in the absence of cell adhesion were determined as reference images. The slight shift between the stressed and reference images was then aligned using the Template Matching plugin in FIJI. The displacement of the fluorescent beads was measured using PIV to create a regular field of displacement vectors. These displacement vectors were then reconstructed using regularized Fourier transform traction cytometry (FTTC) (Sabass et al., 2008) to determine traction forces.

### Keratin Network Extraction and Segmentation of Single Filaments

TSOAX software (Xu et al., 2011; Xu M. et al., 2015; Xu T. et al., 2015) was used for the extraction of filament segments from the recorded images. TSOAX allows quantitative analysis of polymeric network images in multi-dimensions. Its underlying principle has been described previously (Li et al., 2009a; Li et al., 2009b; 2010). Default TSOAX parameters were used with the exception of “Ridge Threshold (tau)”, which was set to 0.008, and “Snake Point Spacing (pixels)”, which was set to 2 to obtain optimal results. TSOAX was executed in parallel instances on the CLAIX/RWTH Compute Cluster (https://www.itc.rwth-aachen.de/go/id/eucm).

The resulting KF segments are defined as polygonal chains with n vertices where each vertex is defined by its corresponding xyz coordinates. These segments, however, are represented individually, not as a connected network. In order to obtain the entire KF network as a single, connected structure, KerNet software was used (kernet.rwth-aachen.de; (Windoffer et al., 2022)). In short, KerNet connects vertices of different segments that are within a defined proximity to form an intersection, called node. These nodes and the segments together form a coherent multi-dimensional map of the keratin network.

### Statistical Analysis

All statistical analyses were performed with GraphPad Prism software. For non-Gaussian distribution, Mann-Whitney test was used. Sample size (*n*), *p*-values and statistical tests are specified in each figure legend. Values are expressed as the mean ± SD. * represents a *p*-value of *p* < 0.05, ** for *p* < 0.01, *** for *p* < 0.001, and **** for *p* < 0.0001. The differences were considered significant when *p* < 0.05. Non-significant differences were indicated by n. s.

## Results

### Low Subcellular Resolution Techniques Are Sufficient to Show the Dependency Between Keratin Flow and Extracellular Matrix Composition

**FIGURE 1:**
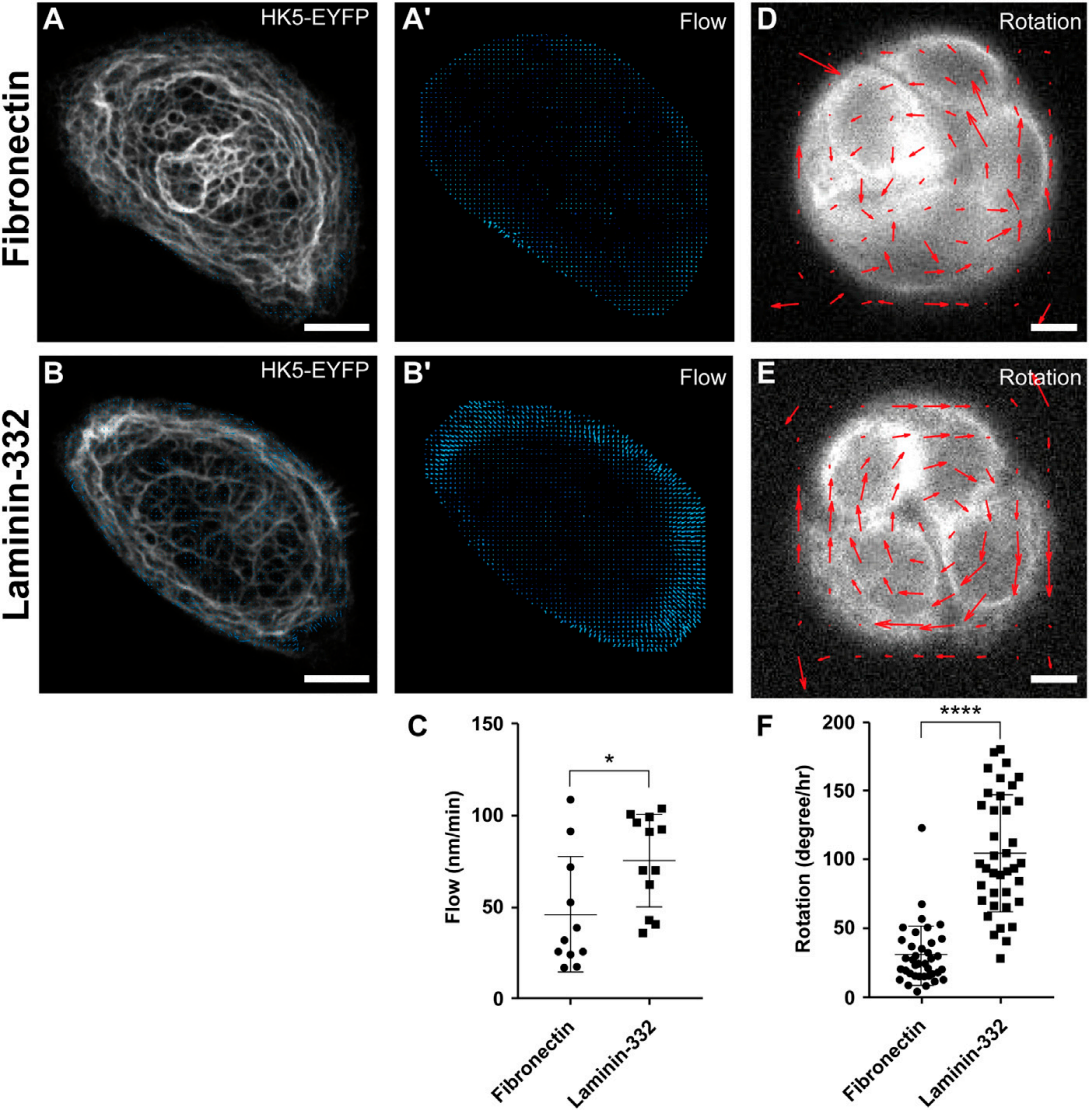
Keratin flow and rotational migration of keratinocytes are dependent on ECM composition. **(A,A’,B,B’)** Show fluorescence microscopy and corresponding keratin flow vectors of HaCaT B10 cells expressing EYFP-tagged human keratin 5 (HK5-EYFP) and growing on D-shaped micropatterns coated with either fibronectin **(A)** or laminin-332 **(B)**. Live-cell images were acquired every 1 min for a duration of 30 min to measure the rate of keratin flow (time point 0 in **(A,B)**; full sequence in Supplementary Movie 1). The local keratin flow rates are shown as blue vectors in **(A’,B’)**.**(C)** The dot plot depicts the results of quantitative keratin flow rate analyses in multiple HaCaT B10 cells. Note that cells growing on laminin-332-coated surfaces exhibit significantly higher rates of keratin flow than those growing on fibronectin. **(D,E)** HaCaT B10 cells were grown on circular micropatterns and fluorescence was recorded every 3 min for 60 min to measure rotational movement (corresponding Supplementary Movie 2). The mean PIVs of rotating HaCaT B10 cells growing on fibronectin **(D)** and laminin-332 **(E)** were calculated and the resulting vectors are illustrated (red arrows) to compare the direction and magnitude of migration. Note that rotation occurred either clockwise or anticlockwise. **(F)** The dot plot shows that that rotation was faster in cells growing on laminin-322- than in cells growing on fibronectin-coated surfaces. Mann-Whitney *t* test, *n* = 12, *p < 0.05, **p < 0.01, ***p < 0.001, ****p < 0.0001. Scale bars: 10 μm.

We have recently observed that keratin dynamics are affected by the coating density of the ECM and by substrate stiffness (Pora et al., 2020). To examine whether the composition of the ECM also affects the rate of keratin flow, we studied cells growing either on fibronectin or laminin-332. We performed confocal time-lapse fluorescence microscopy (1 image/min, 30 min recording time) on single keratinocytes of HaCaT cell clone B10, which produces EYFP-tagged human keratin 5 [HK5-EYFP; (Moch et al., 2013)]. Single cells were seeded on fibronocetin or laminin-332 coated micropatterns with a diameter of 47 μm. The micropatterns were D-shaped to mimic the shape of migrating keratinocytes. The previously described cross-correlation based program CMove (Pora et al., 2020) was used to determine keratin flow rates in the polarized and firmly attached cells. Comparison of the local flow patterns revealed a similar distribution in both instances. Keratin flow rates were highest in the cell periphery (Figure 1A’, B, B’, Supplementary Movie 1) as previously described (Pora et al., 2020). Comparison between the different coatings, however, indicated that the overall flow rates were significantly higher in cells growing on laminin-332 substrate than in cells grown on fibronectin (Figure 1A’, B’, C). The findings support the idea that engagement of hemidesmosomal laminin-332 integrin β4 receptors may be needed for dynamic coupling of the KF cytoskeleton to the ECM.

To find out, whether the different flow rates correlate with different cellular motility patterns, we examined the migratory behavior of HaCaT B10 cells growing on different ECMs. Time-lapse image recordings of the keratin fluorescence in confined HaCaT cells were acquired every 3 min for a total duration of 60 min. Cells showed no movement when cultured on micropatterns consisting of 20 μm wide stripes (data not shown), regardless of the ECM composition. But when multiple cells were cultured on circular micropatterns, they exhibited rotational migration on both fibronectin and laminin-332 substrates (Supplementary Movie S2). This rotational behavior was analyzed using PIVlab (Thielicke and Sonntag, 2021) plugin in FIJI software to track the average velocity of the rotating cells grown on fibronectin (Figure 1D) and laminin-332 (Figure 1E). The resulting vector maps clearly differentiated the migratory behaviors. HaCaT B10 cells displayed a random displacement on fibronectin, whereas HaCaT B10 cells showed a highly oriented rotational displacement on laminin-332. Finally, the rotational rate results indicated that HaCaT B10 cells grown on laminin-332 substrate moved significantly faster than those grown on fibronectin (Figure 1F). The observations indicate that laminin-332 enhances the persistence of cell migration and mandate the investigation of KF network organization at subcellular resolution. The speed of image recording was, however, not fast enough to obtain image stacks, which would allow satisfactory resolution of single KFs or KF bundles in the motile cells and therefore prevented detailed network analyses using previously described image analysis techniques (Windoffer et al., 2022).

### Machine Learning Based Image Restoration Allows Keratin Network Segmentation at Low Resolution Recording

**FIGURE 2:**
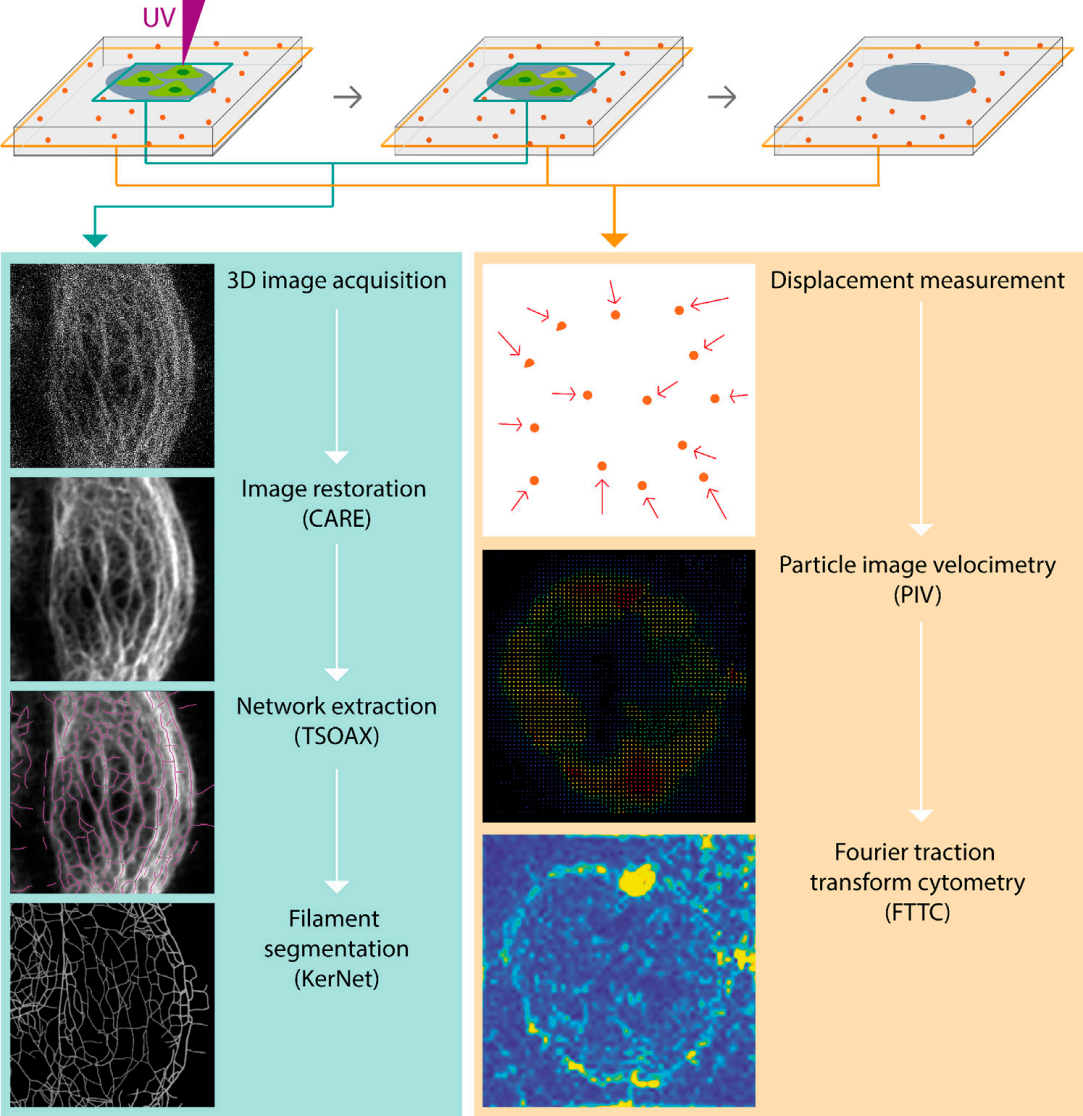
The scheme shows the workflow to analyze complete cellular KF networks in parallel with traction force measurements of cells growing in defined microenvironments. Stacks of fluorescence recordings of fluorescent protein-tagged KFs are processed with the help of a deep-learning based image restoration protocol (CARE) to enhance images to such a degree that they allow complete digital KF network representation. This is done by extracting chains of vertices (TSOAX) that are connected by nodes using KerNet. The resulting numerical representation of single KF segments can be interrogated for various properties such as segment length, curvature and orientation. In parallel, images of fluorescent beads embedded in the underlying elastic substrates are acquired in a separate channel. Displacement of the fiducial beads after cell removal is determined by particle image velocimetry (PIV) to measure their displacement, which can be represented as vector maps (directionality and magnitude of displacement) or heatmaps (magnitude of displacement). The resulting displacement data are then used for the calculation of traction forces by Fourier traction transform cytometry (FTTC).

**FIGURE 3:**
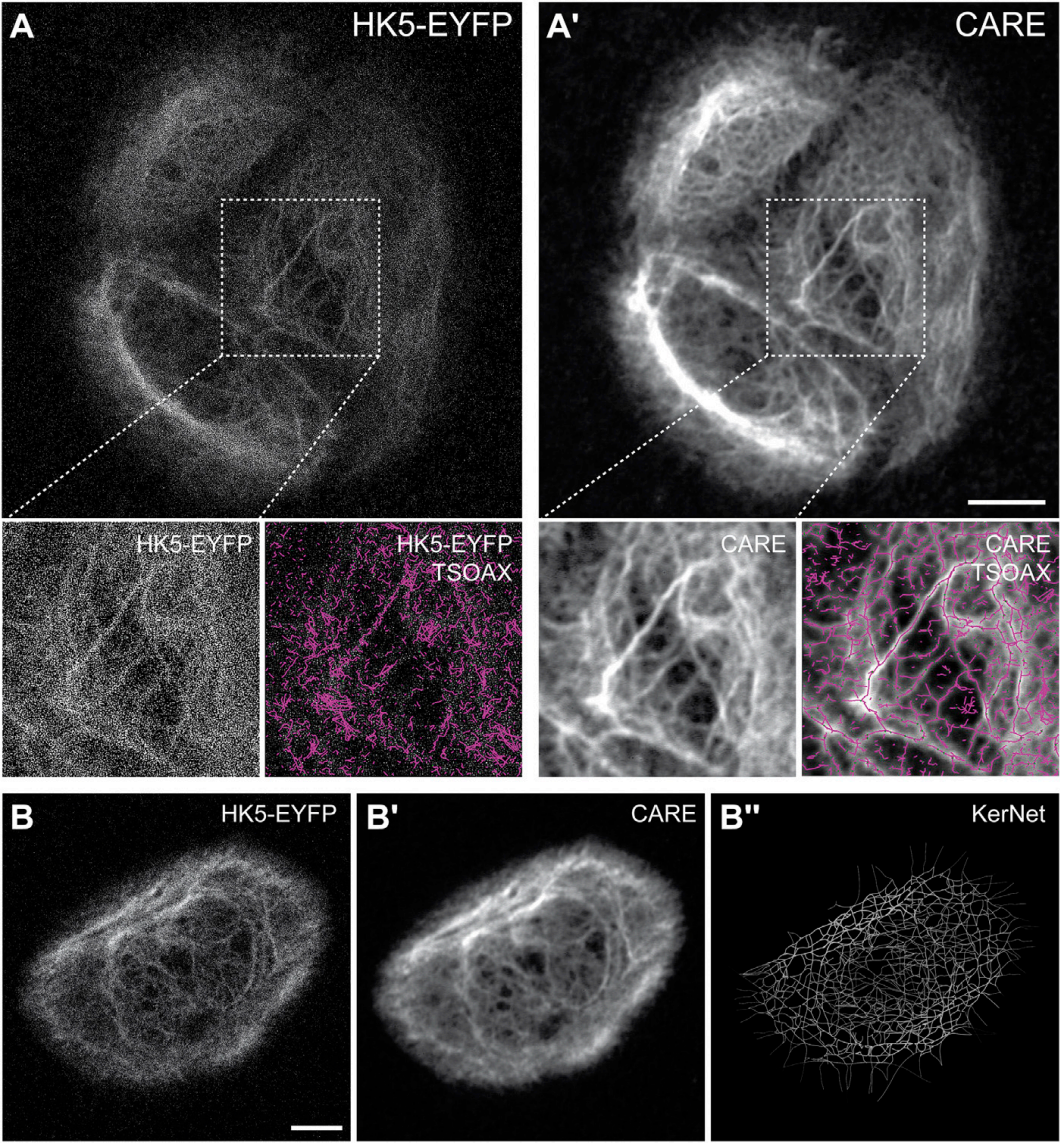
Keratin filament network extraction performance is greatly enhanced for restored images. **(A,A’)** The example shows the raw microscopic recording data (projection view of 20 slices) of the EYFP-tagged keratin 5 (HK5-EYFP) in HaCaT B10 cells **(A)** next to the restored data using CARE **(A’)**. Note that this results in drastically improved KF network segmentation (boxed area). The network segmentation was done with the help of TSOAX using the same parameters for both data sets. **(B-B’’)** Shows an example of a raw maximum intensity projection image stack of HK5-EYFP fluorescence recording in a single HaCaT B10 keratinocyte growing on a D-shaped fibronectin-coated micropattern (left). The restored image is shown in the center and the extracted 3D representation of segmented filaments at right. Scale bars: 10 μm.

Since the microscopic resolution of the fluorescence images were not sufficient to generate accurate segmentation data of the keratin network, we wanted to improve the signal-to-noise ratio (SNR) using machine learning. We decided to apply the recently described Content-Aware Image Restoration (CARE) technique (Weigert et al., 2018). The complete workflow is summarized in Figure 2. To apply CARE, we first had to establish a restoration model. Training of the restoration model was done by using matching pairs of low-resolution images (reduced exposure time and laser intensity) and high-resolution images acquired using the Airyscan mode as “ground truth (GT)” images (Supplementary Figure 2A). 23 pairs of low quality and GT images, each consisting of 5 z-slices, were used to generate approximately 3,000 patches (Supplementary Figure 2B), which were then used to train the keratin network restoration model. The accuracy of the trained model was confirmed by applying the model to untrained images (Supplementary Figure 2C-E). The performance of the restoration model was further validated by comparing the results of network extraction from the raw and processed images using TSOAX and KerNet (Figure 3; cf. (Windoffer et al., 2022)], which showed a significant improvement on the resemblance of the model with the original network structure. The trained CARE model (Supplementary File S1) was henceforth used on all keratin network images prior to their segmentation analysis for the extraction of KF properties.

### Image Restoration Helps to Show That Extracellular Matrix Composition Affects Subcellular Keratin Network Organization

**FIGURE 4:**
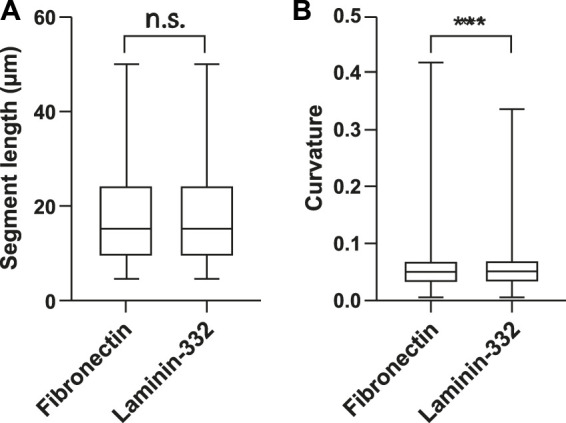
Extracellular matrix composition affects KF segment curvature. The whisker box plots summarize the determination of segment length **(A)** and curvature of KFs **(B)** in single HaCaT B10 cells (*n* = 12 for each condition) growing either on fibronectin- or laminin-332-coated micropatterns. Segment lengths are not affected, whereas the segments are significantly more curved in cells grown on laminin-332 substrate. *n* = 4830 segments (fibronectin) *n* = 6211 segments (laminin-332). ***p < 0.001; n. s., not significant.

With the new tools at hand, we were able to examine the segment properties of KFs in detail and to assess the effects of defined physicochemical niches. To this end, single HaCaT cells were grown on 47 μm diameter micropatterns coated either with fibronectin or with laminin-332 and analyzed. More than 6,000 individual KF segments were examined. We first determined the segment lengths, which are defined as the linear distance between two vertices of opposite end points. No significant differences were detected for the two paradigms indicating that KF network mesh size was not affected (Figure 4A). Next, segment curvature was analyzed. It was calculated as the second derivative of the smoothed tangent vector with respect to the arc length of a given segment. HaCaT B10 cells growing on laminin-332 substrate presented significantly more “curved” segments than HaCaT B10 cells on fibronectin substrate (Figure 4B). The results are compatible with the fact that laminin 332-coating supports hemidesmosome formation and a decreased focal adhesion-dependent tension (Maruthamuthu et al., 2011; Pora et al., 2019; Wang et al., 2020).

### The Novel Experimental Set Up and Work Flow Allow to Correlate Changes in Keratin Network Organization and Traction Force Distribution in Response to Mechanical Perturbation on Defined Substrates

**FIGURE 5:**
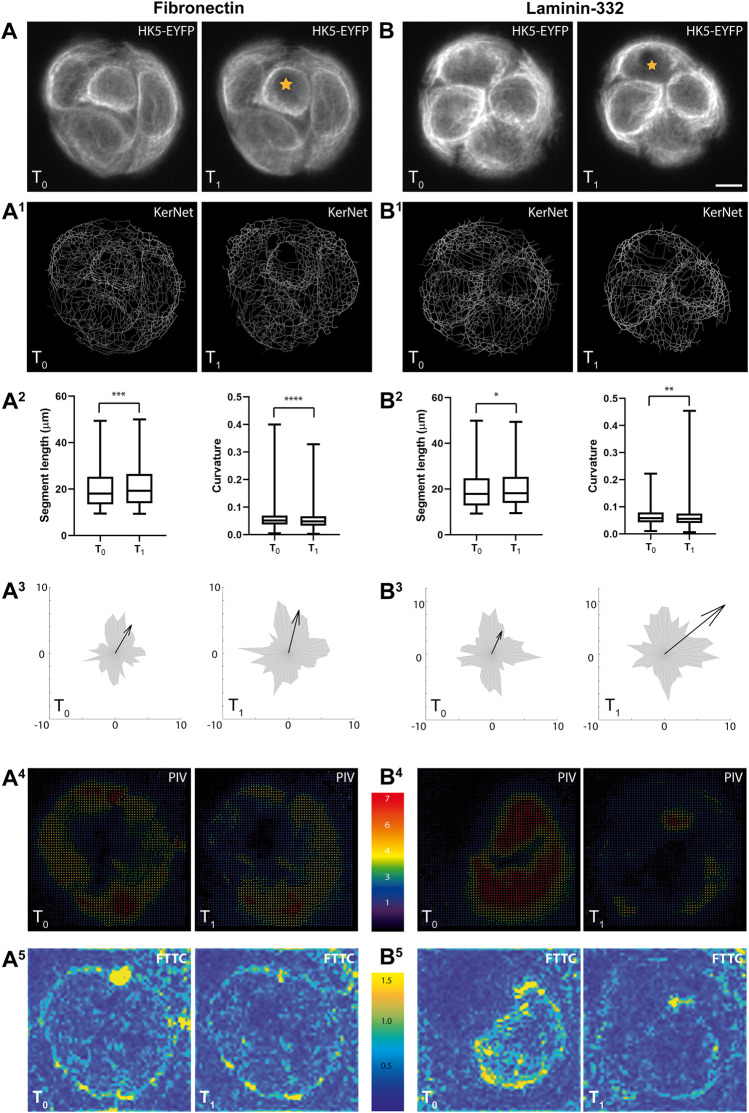
The images illustrate correlating recordings of changing keratin network organization and traction forces in response to local cell ablation and in relation to ECM composition. Small groups of HaCaT cells were generated by using circular micropatterns with a diameter of 47 μm. They were either coated with fibronectin or laminin-332 as indicated. **(A,B)** Show pairs of image projections (20 slices/image) before (T_0_) and 30 min after (T_1_) photo-oxidation of a single cell (demarcated by star). Scale bar: 10 μm (same magnification in all images). **(A** ^**1**^ **,B** ^**1**^ **)** Present the resulting derived digital models of segmented KF networks. **(A** ^**2**^ **,B** ^**2**^ **)** Show histograms of segment lengths and curvatures. Note that both parameters are affected by the perturbation on either substrate. *n* = 1,561 segments (fibronectin), *n* = 1,740 segments (laminin-332); *p < 0.05, **p < 0.01, ***p < 0.001, ****p < 0.0001. **(A** ^**3**^ **,B** ^**3**^ **)** Present diagrams of segment orientations as radial histograms. Vectors are arranged in relation to the center of the micropattern. The arrow represents the sum of all vectors including direction and magnitude. Note that the direction and magnitude of the sum vectors are only mildly affected by the photo-oxidation. **(A** ^**4**^ **,B** ^**4**^ **)** Present the displacement vectors of the fiducial beads embedded in the elastic substrate underneath the micropattern. The vectors were calculated by PIV. **(A** ^**5**^ **,B** ^**5**^ **)** Show the magnitude of the PIV values in kPa representing the traction forces (FTTC). Note the overall reduction in traction forces with only minor changes in overall distribution. Scale bar: 10 μm.

Wounding experiments were performed next to investigate whether and how severe mechanical stress affects keratin network dynamics and whether ECM composition has an impact on this and overall cell mechanics. To this end, HaCaT B10 cells were grown on polyacrylamide hydrogels with fluorescent fiducial beads and circular micropatterned ECM substrates. One of the cells within the micropatterned region was then killed by photooxidation-induced apoptosis. The keratin 5 fluorescence was recorded in multiple focal planes just prior to and 30 min after photoablation (Figures 5A,B) to examine changes in KF network structure. The acquired images were restored and analyzed as described above to obtain a digital representation of the KF network (Figure 5A^1^, B^1^). KF segment length was increased and segment curvature was decreased after photooxidation. This was true for both fibronectin and laminin-332 ECM coating (Figure 5A^2^, B^2^). These findings were somewhat reminiscent of observations in HaCaT B10 cells that were simultaneously treated with latrunculin B and nocodazol resulting in a hemidesmosome-anchored KF network with drastically increased mesh size and increased straight and highly bundled KFs (Moch and Leube, 2021). Whether the two paradigms reflect comparable processes or not, remains to be shown.

We further assumed that the photooxidation-induced cell death introduced an external mechanical stress for the adjacent cells which may trigger reorientation of the KF networks. To test this idea, all detected segments were translated into vector values and plotted onto a single radial histogram (gray polygons in Figure 5A^3^, B^3^). The sum vectors were then calculated (arrows in Figure 5A^3^, B^3^) to assess whether a change in the overall magnitude and directionality of the segments had occurred. These analyses revealed that the sum vectors were elongated. An overall re-orientation, however, was not apparent. Finally, the fluorescent signals of the fiducial beads that were embedded inside the hydrogel were recorded before and after photooxidation to measure the displacement of the elastic substrate. The resulting images were processed using PIV (Figure 5A^4^, B^4^) and were further used to calculate traction forces (Figure 5A^5^, B^5^). The traction force patterns did not show clear-cut alterations in directionality but the overall traction forces appeared to be reduced after photooxidation. Quantification of the traction forces within the depicted areas of interest showed that they were reduced after photoablation (367 Pa versus 321 Pa on fibronectin and 348 Pa versus 268 Pa on laminin-332). The results were also compatible with the notion that traction forces are higher on fibronectin than on laminin coating. We take the comparatively mild effects, however, as an indication for compensatory mechanisms that remain to be elucidated.

**FIGURE 6:**
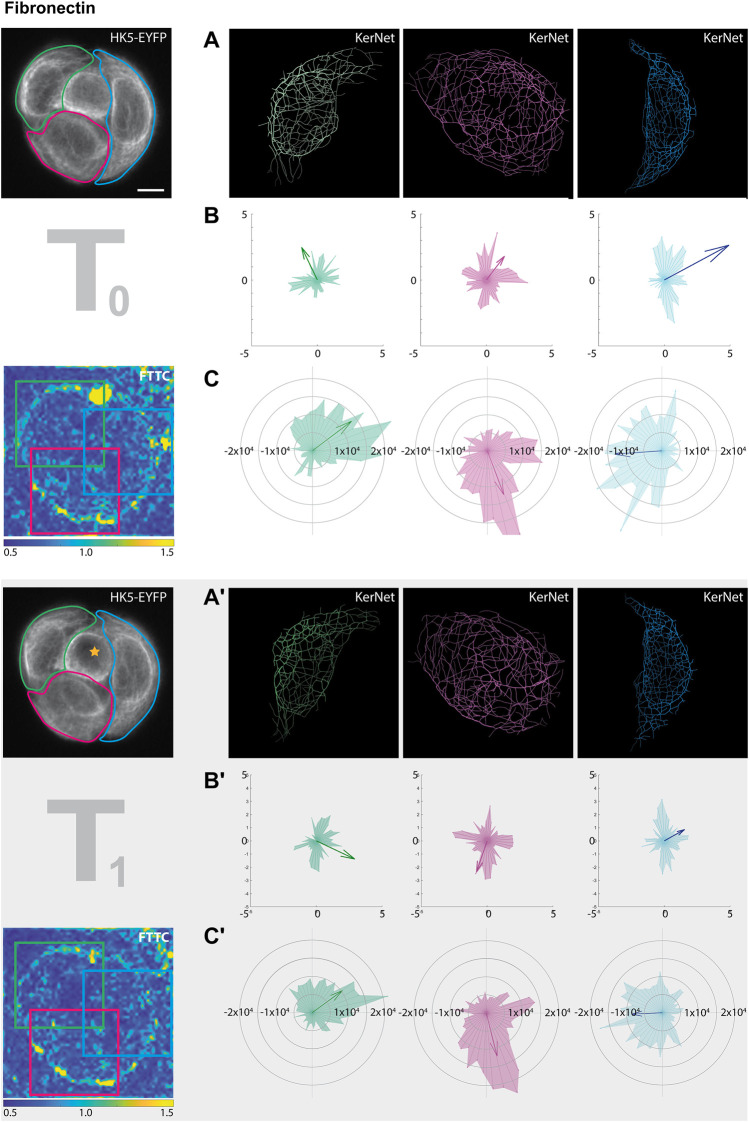
Photoablation affects KF segment orientation and traction forces differently. The data sets for this figure correspond to those shown in the left columns of Figure 5 (partially shown again at left). In this instance, however, the cellular ablation response of segmented filaments was analyzed in single cells within the HaCaT B10 cell group growing on fibronectin. The different cells are color coded in green, red and blue (from left to right). **(A, A’)** Show the digitally segmented 3D networks before and after photo-oxidation. **(B,B’)** Depict the corresponding radial histograms of KF segment orientation and sum vectors in relation to the center of the micropattern. **(C,C’)** Present the corresponding traction force vectors in the areas delineated by colored squares in the FTTC image. They are represented by radial histograms and the resulting sum vectors are illustrated by arrows. Scale bar: 10 μm.

Comparing the segmentation data with the traction force patterns of the entire cell groups provided only limited information. To facilitate a more detailed analysis, individual cells and their surrounding areas were therefore separately analyzed. Figure 6 shows the results for the cell group growing on fibronectin-coated substrate. Similar to the global cell group analyses, segmented filaments of individual cells (Figure 6A, A’) were translated into vectors and represented in radial histograms and as sum vectors (Figure 6B, B’). An overall rearrangement of the keratin network was detected in all cells with the most prominent changes in the green- and red-labelled cells but not in the blue-labelled cell, which only showed a reduction in the sum vector (Figure 6B, B’). A different result was obtained for the traction force. Direction and magnitude were only minimally altered by the photoablation (Figure 6C, C’). Clearly, a more systematic study is needed to carve out consistent response features at the single cell level in wounded microprinted cellular quadruplets.

**FIGURE 7:**
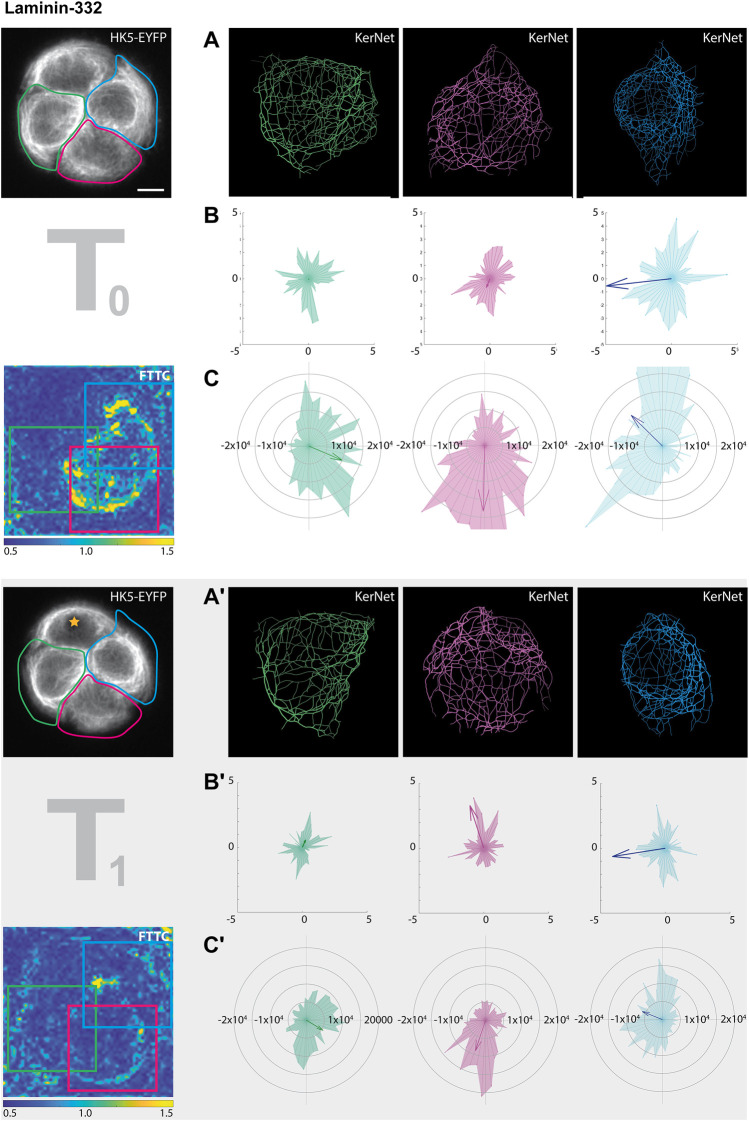
Photoablation affects KF segment orientation and traction forces differently. The data set for this figure corresponds to that shown in the right columns of Figure 5 (partially shown here at left). In this instance, however, the cellular ablation response of segmented filaments was analyzed in single cells within the HaCaT B10 cell group growing on laminin-332. The different cells are color coded in green, red and blue (from left to right). **(A,A’)** Show the digitally segmented 3D networks before and after photo-oxidation. **(B,B’)** Depict the corresponding radial histograms of KF segment orientation and sum vectors in relation to the center of the micropattern. **(C,C’)** Present the corresponding traction force vectors in the areas delineated by colored squares. They are represented by radial histograms and the resulting sum vectors are depicted as arrows. Scale bar: 10 μm.

To test, whether the ECM composition affected the response to photoablation, we analyzed the cell group growing on a laminin-322 instead of fibronectin micropattern (Figure 7). The changes in KF network orientation were less pronounced than those observed for the cells on fibronectin as would have been expected. Again, the changes of KF network orientation were obvious in some cells, notably the red and green cell, but not readily apparent in the traction force patterns. The magnitude but not the directionality of the traction forces, however, were slightly affected.

## Discussion

We describe a method, which allows to simultaneously monitor properties of the IF cytoskeleton at the single filament bundle level and traction forces at subcellular resolution in living cell groups growing in defined microenvironments and their responses to mechanical perturbation.

An important aspect of the setup was the use of machine learning for image restoration. Recent developments in machine learning have helped to develop novel methods to drastically improve spatial and temporal resolution without changing the hardware. In contrast to conventional image restoration techniques, which simply enhance the contrast or sharpness of an image based on the pre-existing data, machine learning allows formation of completely new, predictive data sets by using extensive training of how the image is “supposed” to look. Examples of such machine learning-based image restoration techniques include besides CARE for denoising and deconvolution (Weigert et al., 2017; Weigert et al., 2018), Deep-STORM for STORM super resolution (Nehme et al., 2018) and ANNA-PALM for accelerated PALM super resolution imaging (Ouyang et al., 2018). Use of the image restoration technique CARE enhanced the temporal resolution by a factor of 5 with minimal disturbance of the accuracy of network extraction. Conventionally, up to 5 min of acquisition time using the Airyscan acquisition mode are required to generate a 3D, high-resolution keratinocyte image with enough resolution to accurately detect filament segments for subsequent derivation of a detailed numerical representation. Since the KFs may move as fast as several 100 nm per minute (Woll et al., 2005; Moch et al., 2013), such prolonged acquisition time greatly reduces the accuracy of the exact physical state of the filament network. Use of CARE for KF network restoration allowed reduction of acquisition time to 1 min while preserving the accuracy needed for filament segmentation. The model used in this study was trained using ∼3,000 patch pairs of low and GT images, which were sufficient to accurately bridge the image quality gap between 1 and 5 min of acquisition time (Supplementary Figure S2). To enhance the temporal resolution even further (i.e., reduce the acquisition time of low-quality image to less than 1 min), a new model can be trained with a higher number of raw image data.

The restored images were sufficient to derive precise numerical representations of KF networks at the single filament/filament bundle level from low-resolution recordings and further enabled to assess structural changes of KFs in quantitative terms as described recently for high resolution recordings (Windoffer et al., 2022). Furthermore, we were able to demonstrate that the data on KF segments could be correlated with traction force vectors. It allowed to monitor consequences of perturbing the physicochemical niche (i.e., changes in ECM composition, local cell ablation).

Thus, we were able to show, for the first time, that the ECM composition affects the length and curvature of KFs at the single KF/KF bundle level. This finding further points to the importance of differential coupling of the ECM to the cytoskeleton by different integrin-based adhesion systems. It therefore emphasizes the need to map the dynamic 3D organization of the actin- and keratin-based cytoskeletal filaments in relation to focal adhesions, hemidesmosomes and ECM composition in order to understand the resulting force distribution patterns and the consequences for cell shape and motility. The need for quantitative tools was further underscored by the inability to detect differences in network organization in relation to ECM composition or even after drastic perturbation, i.e. after lethal photoablation of single cells in small microprinted cell groups. Significant changes in KF segment length and curvature, however, could be extracted from the data set with the help of the novel approach, which correlated photoablation with an overall reduction in traction forces. This can be taken as an indication of a cellular response, which became more apparent at the single cell level. But it will be necessary to employ the described methods for more systematic and extensive studies, which would have to include, for example, additional assembly patterns and time points. It will also be crucial to correlate the findings with the responses of other cytoskeletal elements, notably the actin-myosin system. Along the same vein, information on cell-cell adhesions, i.e. adherens junctions and desmosomes, and cell-ECM adhesions, i.e., hemidesmosomes and focal adhesions, has to be provided. It will only then be possible to draw pathophysiologically relevant conclusions on the biological impact. The new tools at hand, however, should afford and considerably speed up this endeavour.

Our work is therefore a first and essential step in characterizing and understanding the cross-talk between ECM and cytoskeletal organization. Understanding the physiology and pathology of the highly abundant and pervasive fiber systems of multicellular organisms both within and outside cells, is important as they make up the fabric of life. They provide interconnected scaffolds with unique local properties that are responsive to local mechanical and chemical cues.

## Data Availability

The original contributions presented in the study are included in the article/Supplementary Material, further inquiries can be directed to the corresponding author.

## Supplementary Material

Supplementary File S1 The file contains the trained model of keratin intermediate filament image restoration.

Supplementary Figure 1 The scheme illustrates the fabrication process of ECM protein-coated micropatterns on polyacrylamide gels of defined stiffness. For details see Materials and Methods.

Supplementary Figure S2 The figure illustrates aspects of image restoration using CARE. **(A)** The matching pair of fluorescent keratin images of the same HaCaT B10 cell, that were recorded sequentially with the same confocal microscope but either with a short scan time at reduced laser intensity (low) or with a longer scan time at higher laser intensity (GT). **(B)** Shows 128 × 128 pixel patches that were selected from the raw images and stored as the training data. They were used to train the image restoration model. **(C)** Shows training data that were used to validate the accuracy of the trained model (CARE). **(D)** Exemplifies, how the generated model was then applied to untrained images to evaluate its performance. Note that the restored image (right) closely resembles the ground truth image. **(E)** The graph shows a quantitative comparison of the noise signals along a diagonal line (from top left corner to bottom right corner) in the low resolution image with the corresponding ground truth and restored image.

Supplementary Movie 1 Time-lapse fluorescence recording of HK5-EYFP in HaCaT clone B10 growing on fibronectin (left) or laminin-332 (right) (see corresponding Figure 1A, B). Cells are growing on D-shaped micropatterns and images were recorded once a minute for 30 min. Local keratin flow rate vectors are superimposed in blue. Scale bar: 10 µm.

Supplementary Movie 2 Time-lapse fluorescence recording of three HaCaT B10 cell groups growing on circular fibronectin- or laminin-332-coated micropatterns (see corresponding Figure 1D, E). Images were recorded every 3 min for 60 min. Note the rotatory movement. Scale bars: 10 µm.
