# Supplementary figures and images for: Combining Image Restoration and Traction Force Microscopy to Study Extracellular Matrix-Dependent Keratin Filament Network Plasticity
Source: Front Cell Dev Biol. 2022 May 11;10:901038. doi: 10.3389/fcell.2022.901038 (PMC9131083; doi:10.3389/fcell.2022.901038)

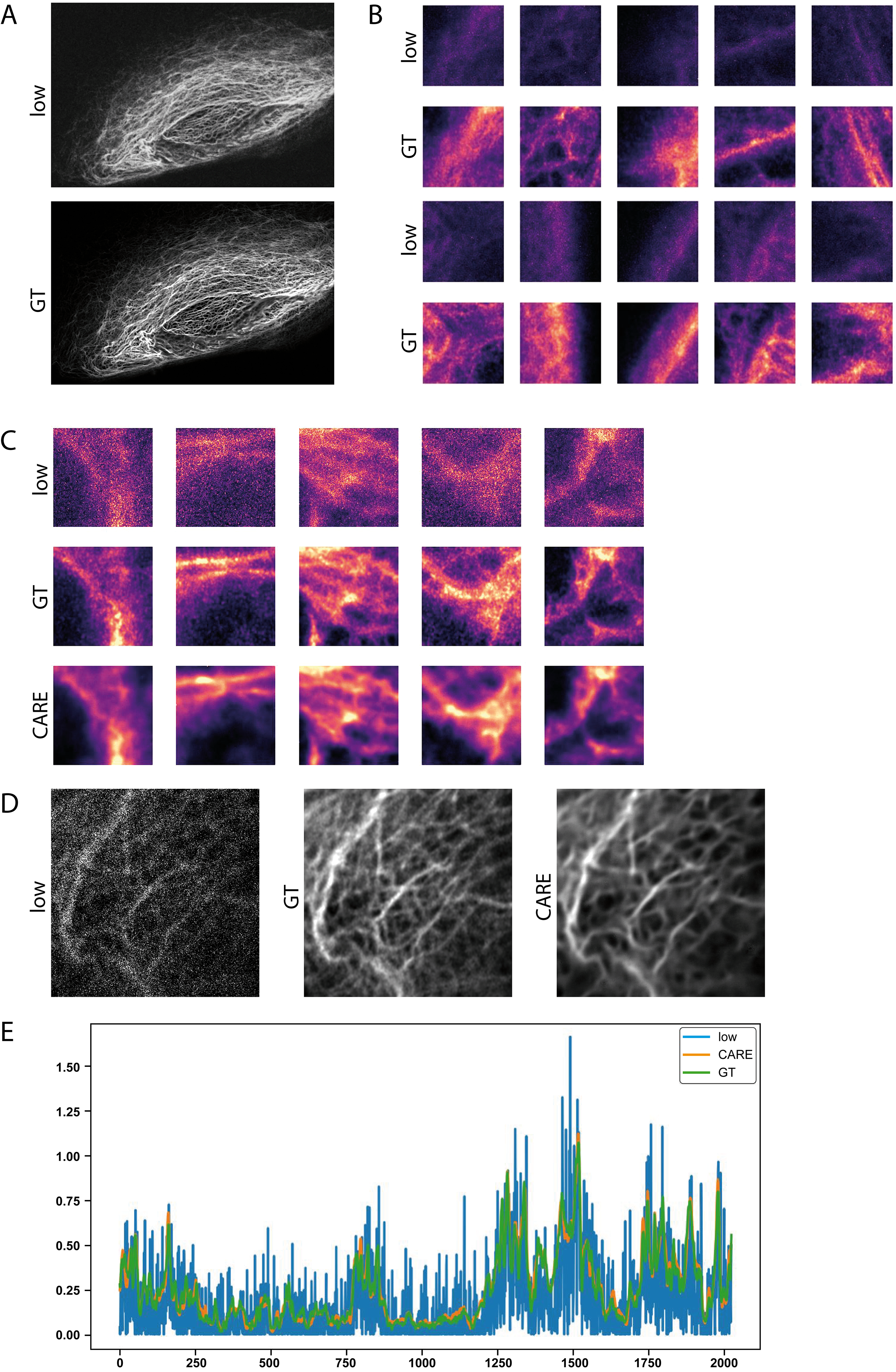

Supplement: Supplementary file 2 [file Image2.tif]
